# Supplementary material for: A whole-animal phenotypic drug screen identifies suppressors of atherogenic lipoproteins
Source: eLife. 2026 Aug 27;14:RP105314. doi: 10.7554/eLife.105314 (PMC13521566; doi:10.7554/eLife.105314)
Supplement: Supplementary file 2 [file elife-105314-code1.zip › JHDL_primary_result/Ketoprofen/Ketoprofen_JHDL_13_010_020_2019_08_16_10_41.pdf]

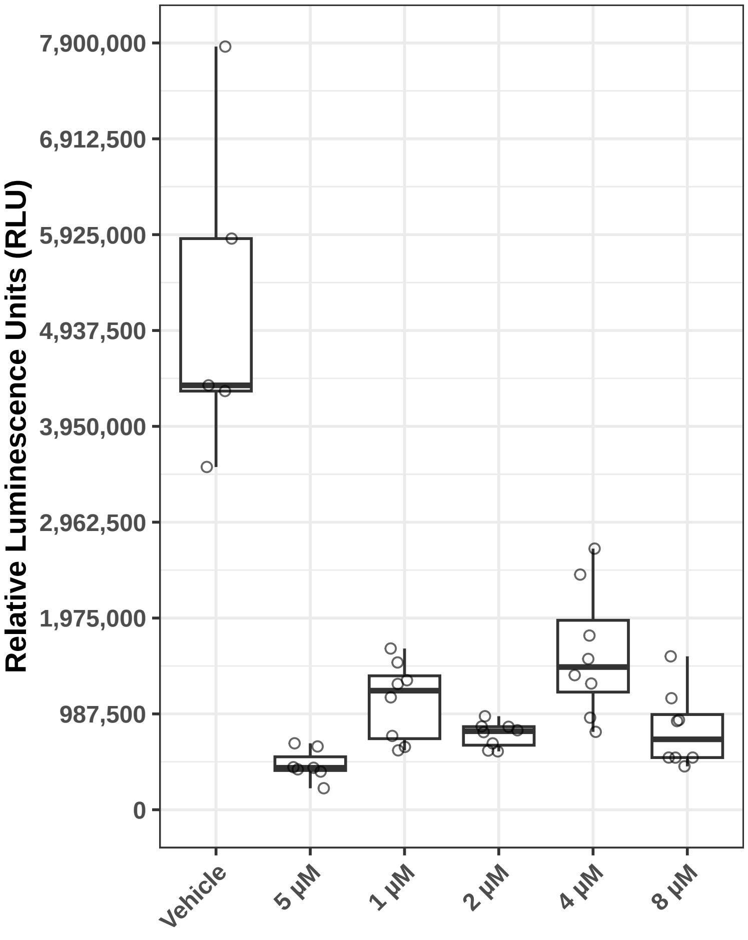

Relative Luminescence Units (RLU)
7,900,000
6,912,500
5,925,000
4,937,500
3,950,000
2,962,500
1,975,000
987,500
0
Vehicle
5 μM
1 μM
2 μM
4 μM
8 μM
